# Entrepreneurs’ ‘Triadic Spatial Paradox’: theorising the hybrid home-based business workspace experience

**DOI:** 10.1177/02662426251410801

**Published:** 2026-02-03

**Authors:** MariaLaura Di Domenico, Darja Reuschke, William Barnes, Cornelia Tippel

**Affiliations:** University of Surrey, UK; University of Birmingham, UK; University of Southampton, UK; ILS – Research Institute for Regional and Urban Development, Dortmund, Germany

**Keywords:** home workspaces, home-based business, entrepreneur, hybrid workspace, paradox, Lefebvre

## Abstract

Responding to literature calls to advance theoretical knowledge of the importance of space in entrepreneurship research, we develop ‘Triadic Spatial Paradox’ as a conceptual model emanating from the creative experiences of entrepreneurs operating home-based businesses (HBBs). Providing a theorised, nuanced unpacking of hybrid entrepreneurial home/workspace experiences, we contribute to entrepreneurship literature by enhancing empirically informed knowledge of neglected space-contexts where paradoxical tensions pervade the lived home/workspace experiences and perceptions of entrepreneurs. The conceptual model emerged from analysing in-depth evidence from entrepreneurs who intentionally set up hybrid home/workspaces. We extend paradox theory whilst embracing Lefebvre’s triadic frame (spatial practice; representations of space; spaces of representation), and advance knowledge about the multifaceted paradoxical experiences of self-regulating, autonomous, home-based entrepreneurs in their HBB hybrid workspace. Using their first-hand accounts and lived experiences of their relationship with their diverse HBBs, we reveal how imaginative engagement with their hybrid workspaces intersects with their perceived, conceived and lived everyday homespaces.

## Introduction

Homeworking during the Covid-19 pandemic notably increased in prevalence attracting interest in the overlapping combinations of workplace and homelife (Felstead and Reuschke, 2023). In the home-based business (HBB), the overlap is an established phenomenon (Kapasi and Galloway, 2015; Vorley and Rodgers, 2014), with entrepreneurs having autonomous, flexible relationships with their HBBs, combined with family roles (Di Domenico, 2008; Reuschke and Houston, 2016; Thompson et al., 2009). Yet, their neglected relationship with the actual HBB workspace should be a key research focus due to shifting entrepreneurial activities and work patterns (Kelley et al., 2012). Homeworking is ‘an integral and stable manifestation of entrepreneurship’ (Kim and Parker, 2021, p.1444); HBBs are not merely a niche, as most small businesses are managed from the home which is often a preferred business space for resourceful entrepreneurs (Baker and Nelson, 2005; Di Domenico et al., 2010; Welter and Baker, 2021). Approximately 59% and 52% of small enterprises in the United Kingdom and United States were home-based prior to the Covid-19 pandemic (Reuschke and Domecka, 2018), with the pandemic encouraging further expansion (Felstead and Reuschke, 2023).

Most small enterprises have no external premises, instead being co-located in entrepreneurial homes or attached premises such as garages, with their characteristics often influencing decisions about homeworking (Reuschke, 2016). Related business activities, like client meetings, occur mainly in the home that might also be used as a base for arranging external events such as client meetings (Mason et al., 2011). While externally located entrepreneurs may bring work home from their formal workplace leading to business/domestic overlap, HBBs are distinct as a phenomenon given they are intentionally hybrid (Sayers, 2010), as they are established at home with no permanent external business location. Despite a lack of research on the inner workspace of the entrepreneurial home, there has been some interest in employee experiences of hybrid, multi-locational workspaces that intersect organisational space and cyberspace with the domestic space (Halford, 2005; Hislop and Axtell, 2009). This has been reflected in the literature on organisational place, and how employees identify with their workplace, along with the effects on this arising from the Covid-19 pandemic, which led to a further increase in remote and hybrid working (Ashforth et al., 2024; Purvanova and Mitchell, 2024; Wheatley et al., 2024). In contrast to such studies engaging with workplace tensions resulting from the organisational control/employee autonomy paradox (Ashforth et al., 2024; Putnam et al., 2014; Wheatley et al., 2024), our study fills a significant gap in the entrepreneurial literature. This has, to-date, largely neglected self-regulating, independent and autonomous HBB entrepreneurs as social actors who relate to their hybrid unilocational workspaces in private homes in ways that affect not only their work patterns but also their entrepreneurial imagination (Kier and McMullen, 2018).

Attention to space and place outside the home in entrepreneurship research (Schmude et al., 2008) has focused on ecosystems, growth-orientation, and scalable regional factors influencing start-ups (Braunerhjelm et al., 2010; Mason and Brown, 2014; Stam, 2015). Virtual technology, multi-location, e-entrepreneurship and mobile space working have attracted attention (Matlay and Westhead, 2005), including their impact on HBBs in the digital economy (Di Domenico et al, 2014; Reuschke and Mason, 2022). HBBs tend to be increasingly technology-linked, as reflected in the homeworking research focus on internet mobilisation and self-employed knowledge workers, emphasising social isolation issues due to increased lone-working in the home and lack of proximity to other entrepreneurs (Daniel et al., 2018). There has also been renewed interest in ‘everyday entrepreneurship’ (Welter et al., 2017), but this has not extended to the dynamics of home workspaces, despite HBB engagement in everyday homespace as a meaningful workspace. There is no equivalent literature focusing on HBB entrepreneurial space-relatedness in terms of intentionally hybrid inner-home workspaces. Our focus on this neglected area of the HBB space responds to calls both for research on everyday entrepreneurial experiences shaping understanding and influencing decision-making (Welter et al., 2017) and on the HBB workspace, given lack of analysis upon the ‘relationship between entrepreneurship and space. . .a challenge to entrepreneurship research and policy’ (Korsgaard et al., 2020, p.699). This neglect motivated the study, as did the need to understand everyday spaces and experiences. This is not only important in developing entrepreneurship theory (Welter et al., 2017) but also in complementing and adding depth to extant research on why entrepreneurs start/develop home-based enterprises (Ekinsmyth, 2011, 2012; Frenckel et al., 2013; Mason et al, 2011; Reuschke, 2019); in addition, it adds to theories on developing entrepreneurial imaginativeness (Kier and McMullen, 2018).

In terms of our theoretical framing, instead of conceptualising HBBs through boundaries or proximity (Ekinsmyth, 2011), to allow greater depth of understanding of HBB homeworking space, we use the Lefebvrian social space framework (Lefebvre, 2000), focusing on the microlevel. Subsequently, we combine this with a paradox lens (Lewis, 2000; Smith and Lewis, 2011) that emerged inductively during the analysis, in order to explore the question: *How does the entrepreneur interact with, and relate to, the hybrid space of their home-based business as a perceived, conceived and lived social workspace?* Lefebvre’s spatial triad (spatial practice; representations of space; spaces of representation) provides a novel approach, in terms of perceived, conceived, lived social space (Lefebvre, 2000), to theoretically explore how entrepreneurs interact with the HBB hybrid home workspace, also seen as a multi-level and socially constructed space (Taylor and Spicer, 2007). To understand space depends on how it is considered, used, experienced and symbolised; this conceptual approach emerged during the course of the research exploring how entrepreneurs articulate a paradoxical relatedness to their hybrid HBB home/workspaces. Thus, we focused on the emergent hybrid and paradoxical HBB enterprise space combinations of work/homespaces from the entrepreneur’s interpretations and imaginings of these spaces. Exploring such paradoxes revealed that these HBB hybrid spaces, imbued with paradoxical tensions, are both inherent to the home workspace and socially constructed.

To explore these issues, we first discuss the Lefebvrian approach to social space to frame the analysis. We then consider HBB spatial hybridity. Based on an empirical analysis of a diverse sample of 34 entrepreneurs in Germany, and their experiences in operating a diverse range of HBBs, we develop the novel concept of ‘triadic spatial paradox’ to enrich understandings of HBB entrepreneurship and the ambivalent hybrid home workspace. This led us to engage with paradox theory, while developing our novel, multifaceted lens from our analysis of the entrepreneur’s relationship with the homespace as a workspace.

## Literature review and theoretical lens

### A Lefebvrian framework for the entrepreneurial hybrid home-based workspace

Lefebvre (2000) advocates a phenomenological understanding of space. It is argued that people as actors intentionally, meaningfully and with agency, create, produce/reproduce spaces for living, working and socialising; moreover, space production is imbued with meaning in everyday social practice (Lefebvre, 2000; Merrifield, 1993, 2011). It can thus, imply social interaction processes with multitudes of conflicting social forces at play. With adornment, representation and appropriation of different spaces by social actors, it reflects how space is a performative product of social actor intents, interests and interactions (Goffman, 1959, 1971, 1983). By studying their perceptions and interpretations of everyday spaces, practices and interactions, relevant aspects of the HBB entrepreneur’s intentions are revealed. A Lefebvrian approach to space sees spatiality as multi-levelled and socially constructed (Taylor and Spicer, 2007) with inherently infused interpretations, values and meanings to which actors relate by using spatial practices in social spaces; they continually change these spaces by making them relational (Lefebvre, 2000; Massey, 2005; Million et al., 2022). This adds to the existing general complexity of the social space (Lefebvre, 2000). These spaces – in being social and physical – encompass perceptions and perspectives of the spatial ‘territories of the self’ of the social actor (Goffman, 1971). Social interaction processes also occur in the space – from which different socioeconomic structures emerge (Goffman,1983), such as in this case, the development of the entrepreneurial HBB located within the home workspace.

The Lefebvrian perspective on space contributes a novel triadic perspective when applied to this inner entrepreneurial home workspace, emphasising spatiality *per se* and the complexity of the relationships the socioeconomic HBB actor has with inherently socially constructed spaces. Lefebvre’s idea of the social construction of space is reflected in three levels in his spatial triad model (Lefebvre, 2000). The first level is *spatial practice* in *perceived space*, and how *actual* space is perceived in everyday life. The second, *representations of space*, is how space is *conceived*, and imagined and communicated beyond direct experiences of spatial practices. The third, *spaces of representation*, is namely the *lived space*, and relates to how space is actually experienced and its meaning to its inhabitants and users. *Perceived space* is a given, often taken-for-granted, literal space; *conceived space* forms in the mind; and *lived space* is a *hybrid space* with the interplay of emerging meanings resulting from combining perceived and conceived space, how it is and how it is imagined.

A Lefebvrian frame has been used before to understand the temporalised rhythm of space in home and everyday life (Goonewardena et al., 2008; Lefebvre, 2004; Nansen et al., 2009, 2010), Airbnb homespace (Farmaki et al., 2020; Stabrowski, 2017), adaptations due to homeworking (Wapshott and Mallett, 2012), and mobile, liminal, third spaces and new ways of working (Cohen, 2010; Kingma, 2016, 2019; Shortt, 2015). It is the context of the multi-usage of spaces to which Lefebvre (1979) drew attention; this has been notably intensified in the late twentieth century with increasing emphasis on Western neoliberalism (Brenner, 2000). A Lefebvrian frame seems germane to first unpack the HBB entrepreneur’s relationship with, and understanding of, the hybrid space combining HBB enterprise and domestic living, to build on related literature on entrepreneurs as social actors (Stuart and Sorenson, 2005). It links to entrepreneurship theories on entrepreneurial imagination in terms of social, practical and creative imaginative cognitive skills combinations (Kier and McMullen, 2018). This informs a realisation of social and business actions and their timing in space (Bjerke and Ramo, 2011), strengthening their HBBs as fulfilment of their entrepreneurial activities evolves in the hybrid home workspace. As space is socially and physically produced (Lefebvre, 2000), this leads us to a novel analysis of how HBB entrepreneurial inner spaces in the home are socially produced. Social actors in this hybrid workspace within the entrepreneur’s home can be compared to both the actors and audiences in the inner territories of ‘theatre stage’ and ‘stalls’ (Goffman, 1959; 1971). Perceived and conceived spaces, as considered in entrepreneurial discourse and research studies (Beyes, 2006; Bjerke, 2010), have been perceived as distinguishable and analytically distinct from representational spaces; the latter having been somewhat neglected despite its relevance to entrepreneurship (Bjerke, 2010).

In terms of our research and the evolving spatial relationships of the social actor in their HBB enterprise, we explore these three types of spaces by also developing our focus on spatial and entrepreneurial imaginative thinking at the microlevel of the HBB site. In doing so, we respond to calls in entrepreneurial literature for more research to ‘contribute to a wider and richer agenda of entrepreneurship studies. . . . A theatre of entrepreneurship has a lot more to offer than commerce and economic drive’ (Beyes, 2006, p. 270).

### The HBB and spatial hybridity: A multifaceted paradox lens

The HBB hybrid space is a combined site (Mason et al., 2011) enabling various functions (Daniel et al., 2018; Di Domenico, 2008) to occur in the dynamic workspace fluidity of the homespace (Nansen et al., 2009, 2010). In the HBB entrepreneurial case, this type of spatial hybridity differs from hybrid workspaces investigated in analyses of employee work experiences when work is, in turn, at home and in an ‘external-office’ (Halford, 2005; Hislop and Axtell, 2009; Wapshott and Mallett, 2012). This literature gives insight into an employee’s spatial experiences, intersecting with the interests and power dynamics of employers and other employees, navigating and negotiating tensions due to the interplay of the differing, often opposing, needs in adopting remote hybrid working (Wheatley et al., 2024). Studies of this genre tend to highlight the role of place in modern, virtual and physical work-places (Purvanova and Mitchell, 2024), and how they have become increasingly uncoupled from the employing organisation for which the work is undertaken (Ashforth et al., 2024).

Whilst certain physical spatio-aesthetic similarities may be present for both the home-working employee and the HBB entrepreneur, inasmuch as they both use overlapping hybrid home-workspaces that are personalised and immersive, important experiential spatial differences involving HBB entrepreneurial issues require further, distinct exposition. This is due to the paradoxes that emerged in this research regarding HBB entrepreneurial autonomy, in contrast to the previously explored organisational control/employee autonomy paradox (Ashforth et al., 2024; Putnam et al., 2014; Wheatley et al., 2024), where there are overarching organisational forces or the presence of an employer. In contrast, the HBB entrepreneur who exhibits much higher levels of agency and decision-making in their work context (Di Domenico et al., 2014), will enjoy more spatial control. Our research thus, takes note of the literature beyond entrepreneurship research in focusing on home and flexible workspaces (Wheatley et al., 2024), in tandem with theories of entrepreneurship, such as those on the entrepreneurial imagination (Kier and McMullen, 2018). In this manner, we offer new contributions to understanding the underexplored HBB context and the entrepreneur’s approach to its inherent spatial hybridity. With this hybridity of space, paradoxical tension levels, dimensions and ambivalences were observed as inherent in the HBB workspace, along with the constructions and interpretations of this space by individuals (Hahn and Knight, 2021).

Understandings of hybridity in entrepreneurship research range from exploring the overlapping identities of so-called hybrid entrepreneurs who, although self-employed, still engage in dependent work (Bögenhold and Klinglmair, 2016) to examining the hybrid mix of entrepreneurial social and economic values in what are mainly social entrepreneurial ventures (Shepherd et al., 2019). Hybridity is also germane in its combined practical and conceptual application to the HBB workspace, as it is embedded in the lived space of the home, a prized, highly intimate hybrid space for the corporeal, personal, imaginative and emotional aspects of entrepreneurship. For the social actor as subject, this may create paradoxical tensions due to the ambiguities of the space, and be managed in a process of continual searching for creative solutions in autonomous working, having actively chosen to co-situate work and living spaces, so as not to require sacrifices in either domain (Vorley and Rodgers, 2014). Despite paradoxes being often seen as synonyms for tensions (Maalouf and Gammelgaard, 2016), not all tensions are paradoxical, though paradoxes create tensions involving ‘opposing perspectives’ (Lewis, 2000, p.760) that can seem incompatible (Poole and van de Ven, 1989). Hybrid HBB tensions tend to be paradoxical, persistent and not easily resolved, but can often be seen as fuelling entrepreneurial creativity (Daniel et al., 2018).

Those tensions co-existing in the HBB hybrid space are examined here, by adding a paradox lens (Lewis, 2000; Smith and Lewis, 2011), to better understand their coexistence, how they are managed and how HBB actors relate to them (Daniel et al., 2018). Through this lens, such HBB space-related tensions are only potentially resolvable; rather, they are seen as coexisting, possibly synergistic oppositions as in organisations (Hahn and Knight, 2021; Hargrave and Van de Ven, 2017). In Lefebvrian triadic hybrid workspace contexts, there may be multifaceted paradoxes, involving creative tensions, as social actors continuously strive to achieve equilibrium (Smith and Lewis, 2011). Lewis and Smith (2014) call for attention to multidimensional tensions in different work settings and a ‘need for a holistic understanding of tensions and cognitive and social influences’ (Lewis and Smith, 2014, p.134). With paradox theorising combined with Lefebvre’s triadic spatial frame, we employ a novel approach to identify how the HBB entrepreneurs experience multifaceted paradoxical tensions in their hybrid home workspaces. We thus enhance current conceptual understandings by analysing the multifaceted paradoxes affecting the entrepreneur’s triadic space-related experiences in their HBB hybrid spaces.

## Method

### Data collection and sampling

The empirical study of HBB entrepreneurs was undertaken in a German region using in-depth qualitative interviews. This is the most appropriate approach to analyse entrepreneurial lived experiences and understanding (Van Burg et al., 2022; Welter et al., 2017) of their everyday practices (Champenois et al., 2020; Steyaert and Katz, 2004). This captures not just what occurs but also the relational aspects, situating the entrepreneurs in context (Thompson et al., 2020; Van Burg et al., 2022; Welter, 2011, 2016). Entrepreneurs operating full or part-time businesses from home, without any external business premises, and working from home for a significant proportion of their time, comprise the target group.

A rigorous sampling strategy protocol was applied, where a systematic address list was developed *a priori*. Entrepreneurs were located in a city centre, with predominantly rented multi-family houses, and in the same region, in a small town on the metropolitan fringe, with mainly homeowners with detached/semi-detached houses. All interview participants in the study areas were recruited via face-to-face contact on their ‘doorsteps’ (Felstead and Jewson, 2000). Due to the size/density of the central city location, every third housing unit was contacted; if there was no reply initially, up to three further attempts were made to establish contact. Properties exclusively used for commercial purposes, student dormitories and retirement homes were excluded either from the address list, if known beforehand, or during the process of recruitment/doorstep contact. In total, 5,603 in-person contact approaches were made, and 102 HBB entrepreneurs were identified; 34 – roughly equally distributed across the two areas – agreed to be interviewed. This approach was generally well-received, as demonstrated by a third of identified HBB entrepreneurs being included in the final sample.

Recruitment of potential interviewees using this approach was decided upon for three key reasons. First, a strength of this method of participant recruitment is that it avoids biases typically associated with snowball approaches used in qualitative studies on HBBs, such as non-identification of potential participants and a tendency for more highly homogenous HBB industry samples (Reuschke and Mason, 2015). Second, as no reliable HBB database exists in the study location, another benefit of this recruitment method was identifying a broad range of potential HBB entrepreneurs in the two areas and, crucially, confirming that they self-define as HBB entrepreneurs. This approach helped address the less visible (Mason et al., 2011; Vorley and Rodgers, 2014) or difficult-to-reach HBB, often characterised as a ‘hidden business’ (Reuschke and Houston, 2016, p.1219). Third, personal contact enabled the build-up of trust with potential interviewees. As a result, questions or any potential doubts of interviewees were clarified from the start, reducing non-response. Drawbacks, principally resourcing for the costs and time incurred, were considered and mitigated as far as possible.

Semi-structured interviews enabled participants to narrate their own story (Larty and Hamilton, 2011). Interviews, conducted in German, ranging from 30 to 135 minutes, were recorded and transcribed, with participants anonymised and pseudonyms for place names. By visiting study locations, all HBB entrepreneurs willing to contribute were included, resulting in a maximum potential sample size of 34, to meet the study criteria from the areas identified. As per Saunders and Townsend (2016), a sample size of approximately 30 interviews meets accepted epistemological good practice standards, as it is approximately twice the number required to reach saturation (Francis et al., 2010; Guest et al., 2006, 2020).

In-depth in-person interviews were employed to obtain rich qualitative insights to most effectively address the research question. In-depth interviews were physically on site whereby visiting the actual HBBs meant interviewers were able to directly observe the HBB space in person. This provided both enhanced appreciation and contextualisation of the narrative accounts and enabled direct observations and verification of the HBB spatial features used to complement the core data of the interviews, enhance rigour and inform the interview analysis. This was the case with 33 of the 34 interviews (the needs of participant #39O necessitated a telephone interview). During interviews, participants were also able to use self-prompts/ elicitation techniques such as physically showing interviewers around their HBB and taking photographs of it in order to facilitate their *in situ* descriptions and meaning-making. This approach led to the development of postscript notes informed by the interviews and observations. These supplemented the interview transcripts, providing triangulation to enable compilation of data tables, thereby enhancing the analysis. These provided detailed information on housing, setting and situation. Table 1 provides the sample characteristics. Business characteristics are provided in Table 2. The 34 participants span 9 industries: health & social care (8); arts (7); events and communications (4); business management (4); IT (3); property (3); finance (2); construction (2); research (1). Participant ages range from 20 to 74 years. Men and women are roughly equally represented.

**Table 1.** Characteristics of study participants (*n* = 34).

Participant #ID	Gender	Age	Business	Household context and situation
04K	Female	48	Research and consulting in the healthcare sector	Living alone (rented flat)
06K	Male	68	Translator	Living alone (rented flat)
12K	Male	54	Artist and photographer	Living with partner & child (rented flat)
13K	Female	20	Online marketing (fitness/health)	Living with partner (rented flat)
14K	Male	48	Consultant for communication & publishing	Living alone (rented flat)
15K	Male	55	IT services and sales/distribution	Living with partner (owner-occupied connected flats in a multi-family house)
16K	Female	55	Childminder	Living together with partner and child (rented flat)
17K	Male	74	Musical instrument maker	Living alone (owner-occupied multi-family house incl. workshop and shop)
18K	Male	29	Rental agency (Start-up company)	Flat share (rented shared flat)
19K	Female	61	Language therapist	Living alone (rented flat)
22K	Female	57	Massage therapist	Living with partner (rented flat)
23K	Female	68	Art therapist	Living alone (rented flat)
24K	Male	48	Stage and costume designer for theatres	Living with partner (rented flat)
25K	Female	57	Office services	Living with partner (rented flat)
26K	Female	59	Software programmer	Living with adult child (rented flat)
27K	Male	55	IT consulting	Living with partner (rented flat)
28K	Female	47	Graphic designer	Living with partner and children (owner-occupied multi-family house)
29K	Female	52	Event management	Living with partner (husband’s multi-family house)
30O	Male	62	Civil engineer	Living with partner (owner-occupied detached house)
31O	Female	66	Management consultant & start-up consultant	Living with partner (owner-occupied detached house)
32O	Female	57	Consulting support for organisational change	Living with partner (owner-occupied detached house)
33O	Male	63	Handicraft with burl woods	House share (rented shared house)
34O	Female	58	Naturopathist	Living with partner (owner-occupied detached house)
35O	Female	53	Journalist and photographer	Living alone (owner-occupied semi-detached house)
36O	Male	55	Data protection officer	Living with partner and children (owner-occupied semi-detached house)
37O	Female	57	Real estate sales and property management	Living alone (rented terraced house)
38O	Male	61	Sales agency/planning office for locking systems	Living with partner and child (owner-occupied multi-family house)
39O	Male	35	Insurance broker	Living with partner and children (rented flat)
40O	Male	43	Production of components for biogas plants	Living with partner and child (owner-occupied detached house)
41O	Male	56	Management training and coach	Living with partner (owner-occupied detached house)
42O	Female & male	46 48	Family-like care centre	Living with partner and children (rented detached house: 2 combined semi-detached houses)
43O	Male	71	Animal and travel photographer	Living with partner (owner-occupied detached house)
44O	Female	48	Childminder	Living with partner and children (owner-occupied multi-family house)
45O	Female	56	Physiotherapist and alternative practitioner	Living with partner (rented detached house)

**Table 2.** Entrepreneurial business characteristics in the sample (*n* = 34), row counts.

Characteristic	Attribute	Total (*n* = 34)
Age of business (years)	0–10	13
	11–20	14
	21+	7
Business Type	Business-to-business	16
	Business-to-customer	12
	Business-to-business and customer	6
Whether clients visit their home	Clients visit their home	25
	Clients do not visit their home	9

### Data analysis

Using Lefebvre’s triadic lens as an initial analytic frame (Lefebvre, 2000), three meta codes were identified: *spatial practices, representations of space*, and *spaces of representation*. Within these, by data reduction, emerging sub-themes and patterns were then identified and analysed. Initial and second coding involved data review for increased analytical rigour and intersubjective understanding (Campbell et al., 2013; O’Connor and Joffe, 2020). Sub-codes and text segments were re-appraised critically to engage with paradox theorising. Data were iteratively, inductively and critically interpreted, moving recursively between theory and data till the coding framework was finalised (Table 3). Theoretical saturation was reached, so as to increase confidence that the number of interviews was optimal for robust analytical insights.

**Table 3.** Analytic frame (theoretical and inductive coding).

Triadic Dimension *Literature-derived Theoretical Meta Code Embracing a Lefebvrian Lens*	Operationalisation (NVivo) *Emergent Thematic Sub-Codes. Phrasal Descriptors of Emergent Themes from Data Reduction and Categorisation/ Data Grouping After Open Coding – Imbued with Paradoxical Elements*	
Spatial Practices Perceived space; socio-physical/material (actual) space. What is done in the ‘workhome’ space and how HBB spaces are used and reproduced in everyday life. How ‘actual’ space is perceived.	• Spatial overlaps and combinations	• Business workspaces/ private homespaces
	• Adaptations	• Orders and routines
Representations of Space How space is conceived and communicated beyond spatial practices.	• Symbolic spatial depictions/ projections	• Future imaginings of space
Spaces of Representation Lived space; social space experienced physically, emotionally, ideologically. How space is lived and experienced, and what it means.	• Spaces of enjoyment, freedom, autonomy	• Spaces of work, personal and family constraints
	• Spaces of inspiration, creativity, focus, and connection	• Spaces of seclusion and isolation
	• Space of proximity, safety, security, and support	• Spaces of disruption and distraction

## Findings

Table 4 provides data examples of evidence from the interviews across all three constructs analysed. Key findings pertaining to the three core constructs are now discussed.

**Table 4.** Data table – examples of coded interview evidence with home-based business entrepreneurs across analytic constructs.

Triadic Dimension: Theoretical Code (Lefebvrian Aggregate Dimension)	Emergent Inductively Identified Thematic Sub-Codes – Imbued with Paradoxical Elements	Illustrative Coded Interview Data Excerpts
Spatial Practices	Spatial overlaps and combinations	■ ‘When it’s very sunny in the kitchen, I sit at the front of my desk because it’s in the shade. . .So I can be flexible, go where it’s comfortable.’ // ‘Well, this is actually my dining room, and this is one of those transitory rooms where I work’ (#23K). ■ ‘. . .people are not that fussy and they do not care if they’re in a nice fancy conference-room or in a . . . in a kitchen (um) and I do not mind and I think I can make people feel much better and more laid back’ (#12K). ■ ‘. . .I also use the garden as a presentation area when I have an open day’ (#33O).
	Adaptations	■ ‘I had to build myself storage space, sheds, workshop, among other things’ // ‘I have obtained a corresponding approval from the local authority here, to have a toilet, everything with me, a guest toilet and something like that it’s all there for. . . You have to have it like the two parking spots in front of the door’ (#33O). ■ ‘Here, there is a complete, independent workroom’ (#36O).
	Business workspaces/ Private homespaces	■ ‘. . .I also lock the bathroom door upstairs. . .That is my private space, it’s no one else’s business which perfume I have, or what type of shower gel I use’ (#37O). ■ ‘. . .my floor is the upper floor, and the patients have the lower floor here’ (#45O). ■ ‘. . .we got the apartment because it was big enough to have. . . a separate workspace for me, that was very important’ (#12K) ■ ‘. . ..I don’t go in there with my customers, but this is the room where only I work, where I spend time the most’ (#22K)
	Orders and routines	■ ‘. . .I hear the tumble dryer beeping just like it is now, then I can go down and empty the tumble dryer and come back. . .I couldn’t do that if my work and private life were totally separate. . .’ (#37O). ■ ‘. . . mostly I sleep rather later and then work at night. . .that’s really like. . .what needs to be done and what I feel like, yes, that’s also extremely pleasant here’ (#18K).
Representations of Space	Symbolic spatial depictions/ projections	■ ‘Wherever I go, I distribute flyers, wherever I am’ (#33O) ■ ‘My lectures were announced in the press. . . then I get further enquiries’ (#43O) ■ ‘I have advertising on my car. . .cards displayed at pediatricians and dentists’ (#44O)
	Future imaginings of space	■ ‘. . .we’re thinking about whether we should even use the flat next door. . .’ (#30O). ■ ‘I’m such a fan of small books, that’s why I wish I had maybe 20 square meters more. . .but to get it we would need to move. . .’ (#27K)
Spaces of Representation	Spaces of enjoyment freedom, autonomy	■ ‘The advantage is natural. The nice thing is I get to choose what I want to do. I don’t have to challenge myself anymore. . . it’s much nicer to work from home’ (#27K) ■ ‘I can decide for myself when to do things. . .I can determine myself when.’ (#6K) ■ ‘. . . when, how, if (laughs) at all, I can decide. . . It’s very important to me, this freedom. So, to be able to set my time myself, to be able to experience my interests, is worth a lot to me, more than anything else’ (#32O)
	Spaces of work, personal and family constraints	■ ‘. . . when my daughters were in puberty and then A-Levels, they needed me and I consciously reduced my workload a bit.’ (#32O)
	Space of inspiration, creativity, focus and connection	■ ‘. . .it’s very important that the kids, who don’t have such a chance anymore to even see a craft. . . so, they sometimes . . .climb on the windowsill and watch us’ (#17K) ■ ‘I do need the peace and quiet here at home’ (#28K). ■ ‘Being at home also gives me the opportunity to work in a calm atmosphere’ (#34O).
	Spaces of seclusion and isolation	■ ‘If you sit alone. . . all day doing responsible things . . . you don’t have anyone to discuss things with, you don’t know exactly what you’re doing . . .’ (#30O). ■ ‘. . . at noon, I have [a] break and I go out, because, at some stage the walls start closing in on me if I just sit here all the time, because I am all alone here . . .’ (#32O).
	Spaces of proximity, safety, security, and support	■ ‘. . .if I get a call. . .in the evening. . .regarding some sort of damage to a property. . .I am sitting here watching television in my pyjamas, I can. . .look for an insurance number so that I can ring the workmen. It’s a lot easier than having to get dressed’ (#37O). ■ ‘You don’t have to drive anymore (laughs). . .just be comfortable at home’ (#31O). ■ ‘. . .If I had an order for a website now, it wouldn’t be an issue with the panic attacks either. You have them for half an hour or an hour and then it’s dissipated again, yeah? And here at home it’s all just OK but not if I’m stuck in an office somewhere’ (#26K).
	Spaces of disruption and distraction	■ ‘. . .when I’m at lunch, on a Sunday, or on a Saturday, and then I actually get a call from some customer like that, ‘but I need so badly, you took a picture. . .’ (#12K). ■ ‘. . .internet connection here is not good. . . it suddenly becomes very unstable’ (#32O).

### Spatial practices (perceived space)

Four themes describe how actual space is perceived: spatial overlaps and combinations, adaptations, business workspaces/private homespaces, and orders and routines (Table 3).

#### Spatial overlaps and combinations

Spaces defined as primarily homespaces are also paradoxically combined in use for interactions with clients. #12K held meetings in his ‘more comfortable’ living room and kitchen, reflecting how he wishes to present a more private social space to clients. Hosting cultural sector clients, living spaces are chosen to represent him as an artist and photographer, adorned with artwork and books they are much ‘more beautiful’ and ‘artistic’ than meeting rooms. The paradoxical hybridity of spatial overlaps/ combinations is reflected by #14K saying his ‘living-room’ is ‘not really a living-room’, as both a private space and a ‘sort of a room for my work, for my visitors’. #35O describes how the kitchen/living-room takes on a temporary guise as a room especially for long meetings, as it is comfortable with ‘an all-purpose space with coffee’, merging her hosting role with that of professional journalist and photographer. #34O, a naturopathic, treats clients in her garden, using kitchen, bathroom and utility-room items to work with allergy patients, and kitchen for dietician work to show clients how to bake, as ‘being able to do that here is an advantage for me’. #41O uses his garden and terrace for management training and coaching workshops. His ‘open-house’ policy is; ‘this is their home for the next 4 days now, I say make yourself at home and feel at home’. Where such clients have full entry to the homespace but are aware of its other uses, a paradox is that as clients they access them as workspaces, not homespaces. Despite this combined usage, for #41O, when workshops are over, or if a client returns, it is as if the space is transformed from open-house to private home or vice versa.

The notion of transformations, through spatial re-combinations and practices, is thus germane in our analysis. Some participants said how they undertake considerable work to ensure their home is ready for clients, especially when clients are ‘patients’ such as with the naturopathist and physiotherapist, who transform the household environment to meet hygiene regulations (#34O, #45O). These transformations take place with prior planning to ‘set the stage’ for the clients before they arrive: ‘So we don’t have to worry. . . our schedule is professional’ (#41O). Work undertaken to prepare for clients includes cleaning, catering, food shopping and preparation of the seminar room with ‘props’ like chairs, tables and flip charts set out as appropriate for the group. ‘. . .I’ve got a plan up there. . . there are four participants with a table or ten participants in an open circle of chairs’ (#41O).

Although he also calls it an ‘open house policy’, and presents it to the clients as such, at the same time #41O installed strict rules relating to organisational concerns: ‘So that was the goal that I set myself . . . the goal, that I’m so concerned about, the organisational things. . .’ (#41O). Participants in the workshops are expected to follow the rules; on Wednesdays, they are not allowed to remain in the evening; on Thursdays, they are welcome to stay later; on Fridays, they are not only expected to stay in the evening, but can bring others, with former participants being able to join. When they have gone it ‘doesn’t always look great in the morning and then I’m glad (laughs) . . . Clear the decks!’ (#41O). #14K’s flipchart is also returned to the basement after client meetings, as he does not like to see it when not working. This strategy helps him feel in control, continuously negotiating, renegotiating and redefining the hybrid, paradoxical homespace-workspace through judicious use of such props. Indeed, mental shifts in perception to transform, or alter, the definition and usages of overlapping or combined physical spaces occur, aided through the use, and then removal, of the props or other representations, and such spatial practices were found to be key to the way in which the HBB entrepreneurs perceived these spaces in their ever-changing home/work contexts.

#### Adaptations

Some participants moved to a new location to accommodate their HBB; others extended their homes for the business, or divided, converted or adjusted existing rooms to ‘better set the stage’ for their HBB clients. Of the seven participants who moved because of business needs/ activities, they also noted benefits for their personal life and space. Thus #40O built new premises to have a production hall and home together whilst #41O moved to be able to shift all workshops and seminars to his home setting. These new properties are now sufficient to act as both workspaces and family homes, although each must still adapt to accommodate the other. The same was described by the six participants who extended their homes to operate their HBB better. Thus, #12K rents an additional room below his property to use as a conference room. #15K bought two additional adjacent flats and linked them to his own to allow sufficient workspace. #33O built a workshop in his garden, creating added space for his HBB. #42O also linked his home to the adjoining house, while #44O joined hers, and the adjoining apartment, to create space for her HBB childminder business.

Whilst these participants made significant changes to accommodate their spatial working practices, other less intrusive adjustments were evident, requiring more input of entrepreneurial imaginations, with rooms once used for domestic purposes being repurposed to workspaces. Thus, #33O’s front room was changed into an exhibition space. #25K put office equipment in her living room. Homespaces not actively used are reused. #34O made her former basement party room into a treatment room. #41O converted his garage into a seminar room. Five others converted unused bedrooms to workspaces (#22K, #32O, #35O, #40O, #43O). Yet, the spaces are perceived to be fluid with the potential to be converted back to domestic use. Five participants had adapted their workspaces for personal, family and domestic uses; three changed workspaces back to fulfil family needs. #28K had used an adjacent apartment as her workspace; however, she wanted her father to be closer, so she moved her work to the kitchen/dining/living area so he could use the next-door apartment.

#### Business workspaces/private homespaces

In terms of workspaces in their homes, participants designate as business workspaces their ‘home-offices’, exhibition and sales rooms, meeting-rooms, guest/client facilities, studios, workshops and storage spaces. The home office, an exclusive space in the home for computer work and paperwork (and some meetings), is the most prominent space mainly kept separate with 20 participants having such an office.

The number of participants enjoying the ‘luxury’ of having a home office differs significantly between the two locations, linking up with type and size of homes. Compared to 13 of the 16 in the suburbs, seven of the 18 participants in the urban area had home offices, due mainly to a central city location and apartments not being easily subdivided. Those in the small town live in detached or semi-detached houses, allowing them to divide their rooms to have meeting-rooms, guest/client facilities and storage, and so home offices are more prominent in this less densely populated area than in the city where use of hybrid spaces for lone work is more common. This also allows full use of spatial imagining in repurposing living spaces as offices. A wish for, and reality of, a home office in the small-town links to the entrepreneur’s age, suburban lifestyle and self-definition. This may imply different generational attitudes to the paradox of using different spaces to represent the separation of worklife from homelife, while facilitating the entanglements and connectivities deemed necessary between these two arenas. Both the female (#13K) and male (#18K) entrepreneurs in their twenties living in the city in rented flats had no designated workspace, sharing space with either a partner or flatmate. This contrasted with the clearly designated workshop spaces prized and enjoyed by the two entrepreneurs in their seventies, who lived in more spacious owner-occupied premises either alone in the city in the case of the musical instrument maker (#17K) or in the suburbs with his partner in the case of the animal and travel photographer (#43O).

Half of those with home-offices said clients can enter if they permit it. More than half (18) of the participants bring work into the private homespace. Boundaries must be maintained for others, with the home office a secluded, private space to which they escape, an extension of their personal space. Three specifically comment that customers, clients and business stakeholders are not allowed into their home offices. For #22K, it matters not that it is a mess, as it is closed to clients. For #36O, only his wife enters at certain times. An alternative practitioner/physiotherapist, #450, lives with her partner in a suburban detached rented house. She stressed ‘the office is supposed to be confidential, no one is allowed in there’. No patient is left alone upstairs or able to enter her confidential workspace without her presence. For another six, home-office space was important, an ideal way of separating work and home. For five, desiring key space for a home-office is why they moved, or extended their homes, depicting it as ‘core’ (#12K), ‘base’ (#31O), ‘cockpit’ (#41O). While this suggests its centrality to the HBB success, it is not for everyone. #42O’s home office is seen as ‘too small and chaotic’.

‘Workspace’ or ‘business space’ was explicitly mentioned and described as separate from ‘homespace’ or ‘private space’ by 21 participants. Those with clients visiting the home emphasised spaces as ‘off-limits’ to clients. Those in the suburbs noted that their more flexible space significantly determined their HBB spatial arrangements and practices, with more from that location, compared to the city-based participants, referring to ‘private space’ or ‘homespace’. This was despite those in both locations displaying ambivalence about a client’s presence in their home. Three participants did not welcome clients into their homes due to privacy concerns and wished to offer a more professional impression. #18K prefers to meet clients outside his home and instead meets them in restaurants and cafés, as a more neutral ‘third space’, although he sees this also as very much a spatial extension of his own space and homeworking. When his home office was in an annex, #30O met clients there. Yet, after moving it next to his bedroom, left open for his dogs, he changed his practice, perceiving home as too private for customers and clients. #16K does childminding 3 days a week at home but keeps a barrier between her own biological children and those for whom she cares, putting away their toys, cots and equipment at the end of each day, and bringing out those of her own children. She refuses invitations to birthday parties from parents to keep them only as clients, rather than friends. Yet, participants have self-imposed rules they choose to follow, or not, if/when they wish to keep or break them as autonomous entrepreneurs. #37O has clients nearly every day in her four-storey house with workrooms spread over all floors and rules invented to structure access to specific areas of her house. Her rule is to meet clients in a meeting room on the first floor. She can choose to take long-time trusted clients downstairs to a living room or garden, but not ‘unpleasant clients’, who go straight to the meeting room. Clients use the guest toilet on the ground floor; she locks the private bathroom upstairs: ‘That is my private space, it’s no one else’s business which perfume I have, or what type of shower gel I use’. In contrast, some participants allowed their clients more access to private spaces and even to stay overnight in their homes. #41O can allow clients to sleep in his spare rooms during multi-day seminars. #24K, when working with visiting theatre directors, permits them overnight stays.

The theme of private space, combined paradoxically with transparency, was reflected on by #17K who runs a musical instrument shop, workshop and concert business. For him, all are welcome and invited to enter or watch. He gives open tours to children, students, clubs and societies. His city workshop can also be seen from the outside through large glass panes. Many stop to watch the work through the window as passers-by, accessing the inside of his workspace visually by means of the window but, unlike clients or visitors, not playing an active part in the workspace and its props, as paradoxically they are part of it, but separated from it by the window glass. Yet in the evening, if he wants to work alone in a quiet private atmosphere, he can choose when and if to close the louvered blinds to cover the window. In contrast, 12 participants hold meetings in the home of a client, or use other third spaces as well as the home, including networking events and public spaces such as cafés and parks, and 15 describe material artefacts they take with them from home if needed to work in external spaces. Predominantly, these are laptops or similar electronic devices. Participants say they are able to take parts/ elements of their workspace with them, and so create a virtual workspace. In such external spaces and multi-locations, there remains a strong relationship to the ordering of practices within the HBB, as both are influenced by the homespace rhythm.

#### Orders and routines

Among the participants, 13 have specific working hours in order to structure their day into defined periods, or starting points, for their client appointments. #30O describes starting at 5.30 am and working until 8.30 am to ensure getting a lot of lone work done before clients start to call. #15K defines 5.30 pm as the boundary ending his work day, leaving his phone behind if he goes out because ‘then it’s time to live’. Alternatively, children govern work hours of #28K, with her typical day involving working until noon when her children are at school (typically morning only as per the norm in most German schools).

Similarly, #36O tries to stop his formal working day in his data protection business, where he mainly does paperwork, when his children return from school, with this being a time boundary marker, though there still remain ever-present different markers and symbols within the home workspace, a reminder of the beckoning of other priorities. Thus, markers and symbols for #36O include ‘many, yes, slips of paper on which something is noted, which I have to process’ along with household ‘helpers’ reminding #36O of housekeeping duties ‘such as working with the dishwasher, the washing machine’ and ‘according to what the customers have to do in the afternoon, I have to ensure that the sports equipment is laid out, etc. and such’. Markers and symbols include those objects that are present in his workspace that ‘I can put down here whatever I want, so that I feel well’, reminding him of ‘nice things’ while he is working as; ‘I have everything there which is fun for me, from my Pippy Longstockings picture, to a small Linux penguin, to a lighthouse, whatever, also a ship’. Other participants also have ‘feel good’ markers and symbols in their home offices of their hobbies or quality of life away from their business.

However, #27K and #41O keep their business and private lives separate on larger temporal scales. #27K takes days off for hobbies. #41O takes 3 months off each year as ‘when we’re here [home]. . .it’s our world of work. . .and timeout takes place elsewhere’. Weekends or nights were explicitly kept free by 11 participants, while 22 adopted mixed approaches with flexible temporal/spatial strategies allowing switching between work and domestic activities. Nine participants paradoxically have elements of *both* flexible and fixed temporal strategies in their typical daily routines. Thus, there is not a completely rigid division of time for #36O as ‘the morning consists actually of work plus a little housekeeping, plus a little bit with the children in most cases, if nothing in particular is pressing’. For most, this is a welcome flexible time for family/social interests. #45O reflects on her malleable HBB approach, and how she pivots easily to do other things if clients cancel at short notice: ‘I could do anything else I would do if I were at home’. Changes of space in time are key. #14K discusses cooking as a break from ‘very brainy professional activity’. #35O’s HBB provides space to connect creative work with home to relieve work stresses. She walks the dog or does housework if unable to do creative work but works long hours into the night when creatively engaged or with deadlines. Most are positive about their spatiotemporal flexibility. Others are ambivalent. #29K struggles to stop thinking of work. #37O feels they are ‘never stopping working’.

### Representations of space (conceived space)

The second element of Lefebvre’s spatial triad is ‘representations of space’, encompassing how the hybrid home workspace is *conceived*, and also communicated, beyond any direct experience of spatial practices. This is most prevalent in our HBB study in terms of symbolic spatial depictions and projections, as well as in creative future imaginings of space (Table 3).

#### Symbolic spatial depictions

Our participants use symbols to depict the HBB space to self and others, such as #17K’s workshop window which he considers symbolic of openness to the outside, but when obscured with blinds to hide his workspace, it paradoxically also symbolises seclusion. These participants try to show their businesses as attractive to prospective customers through marketing and business strategies. #33O said: ‘Wherever I go, I distribute flyers, wherever I am’. They depict them in flyers (#22K, #26K, #29K, #33O), newspapers, brochures, on cars (#22K, #31O, #43O, #44O, #45O), and through business cards (#22K, #31O, #44O), websites (#24K, #31O), and in networking groups of other self-employed HBB practitioners in their business sphere (#22K, #44O). #43O courts publicity by giving lectures on his HBB: ‘My lectures were announced in the press. . .then I get further enquiries’.

Physiotherapist and alternative practitioner #45O is an outlier compared to the other HBB owners who are more trusting of those entering their home, as, although they may also have rules about access to their homes, they are not controlled in such a strict material way. Thus, despite advertising her services, #45O values her anonymity and business invisibility, not publicising her location or details of her home workspace. She installed a strict regime to control patient access to her home, supported materially by an intercom hidden in her cellar where her treatment room is located. It symbolises her control of all entrances and exits, as she elaborates on: ‘an intercom system for entry, you can see it here. I can look up what’s on the street, if it weren’t so dark right now. I can see which car is there, I can see that’. She avoids telling the local authority about her business use of homespace by not submitting applications that could be rejected due to residential location and hides her business from neighbours. ‘I come from a village where the neighbours always knew everything. And I did not want them to know who exactly is my patient, who is my friend? I thought it was more important to be anonymous’. She stresses micro processes that help her plan her working day routines, also symbolising her control over them. It shows ambivalence as she said she developed these routines to make every patient feel special, but does not trust them, seeking control not only over their access to other patients, but also to private areas of her house. Consequently, #45O ideally does not want a client to be aware of other patients: ‘This is my ideal life. Then no patient sees that there was one before. Everyone thinks, I’m the first, I’m the only one, I’m the most important, I’m the best patient’. She imagines they do ‘not want to know that there are other patients who might have been here too. And I don’t want it either’. Yet if control breaks down, tensions and feelings of conflict and anxiety can result if someone has to wait for another to finish, although she reassures herself that: ‘rooms are reasonably soundproof. But I know from other physiotherapists practices, you can hear a relatively large amount. . . not nice when the patient sits directly next to the wall and listens in. . . .fortunately that doesn’t happen here’. Sturdy cellar walls protect her privacy, whereas they ‘usually only have plasterboard walls’.

#### Future imaginings of space

Of the seven participants who drew mental pictures of their future imaginings for their HBB workspace, they included descriptive plans for altering room layouts or other spatial adjustments that they envisage. They link and intertwine their plans for spatial expansion of the home, or a new location, with plans for business development and growth. When #18K finishes studying, he plans to potentially expand the business model, which might involve moving premises. #30O noted: ‘I’m thinking, or we’re thinking about whether we should even use the flat next door. . . .’ #26K does not plan to move as her present home is a place of security for her, where she wants to gradually grow the business. At one point, she had to restart the firm with no investment after bankruptcy arising from her husband’s debts. #40O is thinking to grow the business and expand the HBB complex to accommodate growth but is still concerned about market conditions: ‘we’ll dial it up a little, maybe expand the business model a bit, (um) maybe take some bigger portfolios with us and that’s supposed to be long term’. Yet, two are content as they are, and do not want to grow their businesses or change the spatial arrangements of their homespace: #37O sees her business as the perfect size: ‘I can run it from home and there is enough work to keep me going until I retire’. She reflects: ‘I don’t want my business to get any bigger. (laughs). No, I have definitely reached my limit now. . .If my business got any bigger. . .doesn’t necessarily mean less work and more money’. #27K is even scaling his workspace down as he prepares to end his professional career.

Retirement is imagined and discussed mainly by those actively planning it. #31O intends to turn the space she currently uses for work into an apartment when she retires. #38O intends to move into the basement he currently rents to tenants when he retires, and in turn, rent the first floor where he lives and works. On retiring, #41O plans renovations to replace worn furniture in his seminar room. #19K imagines herself moving away upon retirement, not taking the ‘whole thing with her’. #27K plans to move his book collection to a bigger apartment to have a dedicated library: ‘I’m such a fan of small books, that’s why I wish I had maybe 20 square meters more, because. . .I could use my own library again. It’s not a bad idea, but to get it we would need to move’. #27K cannot have a dedicated library for his book collection in his present location due to the space being used for business. Even scaling the business down with the prospect of retirement, he must juggle the available space in his rented apartment and is reluctantly considering moving to a bigger one, though he envisions that this will create some tensions and new challenges. The example of #27K’s value of his books and desire for a dedicated space shows how this imagined space is imbued with meaning, affecting his social practice. An example of ideals embedded in a conceived space is #17K’s imagined impact of his window space on local children: ‘I think it’s very important that the kids, who don’t have such a chance anymore to even see a craft. . .can see that here. . .climb on the windowsill and watch us’. The paradox is that this combines with a desire for privacy to undertake his work, balanced but never fully resolved by his deliberate use of window blinds.

### Spaces of representation (lived space)

Here, the third element of Lefebvre’s spatial triad, ‘spaces of representation’, encompasses lived space and how this hybrid space is paradoxically experienced (Table 3).

#### Spaces of enjoyment, freedom, autonomy; and work, personal and family constraints

Depicting HBBs as spaces of enjoyment and freedom is a key theme permeating these participant narratives. They see freedom as choice, flexibility, balancing work with family and homelife, the home workspace allowing work and family to flow; and working in different homespaces: ‘I can be flexible, go where it’s comfortable’ (#23K); reducing workload if necessary for childcare and family duties or personal needs (#32O). #14K considers ability to ‘carefully cook’ lunch in the kitchen as a ‘nice thing’; for #15K lunch with his wife makes ‘the whole thing worth living’; #30O talks of his conference-room, now a billiard-room decorated with motorcycle pictures representing freedom to go from work to his motorcycle trips or to play games as, ‘It’s very important to me, this freedom’. Again #30O feels free at home to work in the morning in pyjamas and at night with a glass of wine; and #35O’s HBB ‘offers unending possibilities’, making her feel fortunate to work at home throughout her professional career.

Although such notions of enjoyment and freedom from homeworking permeate narrative accounts, with home described as a ‘pleasant’ place for #14K to ‘work best’, and for #36O to ‘feel well’, this is tempered by the ever-present juggling of demands of work and family commitments. #24K enjoys freedom to organise work around his personal life, resulting in a ‘good, healthy balance’, although he admits his freedom is constrained by work demands, and workload governs his available time. For the entrepreneurs, a sense of freedom is enhanced by their HBBs giving them a sense of autonomy and control over business, time and private life, although they continually balance work, personal and family demands. #14K describes feeling liberated by no-one ‘looking over my shoulder’. Likewise, #24K has ‘free reign’ where ‘no one controls things’ and #27K has a freedom not to ‘depend on someone’s approval’. Yet, they still have to take responsibility for managing their commitments in terms of space and time. This is illustrated by #35O, who explains that freedom means she can take breaks when having writer’s block, as no one else, but herself, sets her strict rules or daily aims and deadlines. The control over time and space, despite paradoxical tensions of their workload and personal priorities, remains liberating through the association with their lived space.

#### Spaces of inspiration, creativity, focus and connection; and those of seclusion and isolation

For #17K, the instrument shop owner, his HBB workshop is a space of inspiration for him and ‘for the children’ who visit or peer in the window to watch his craftwork. This reflects his wish for social connectivity to inspire others while relishing his solitude. Explaining his lived workspace spatial representation, he is: ‘. . .quite comfortable in my own room. . .listen to my own music. . .concentrate myself on the handicraft work. That was always my dream.’ Creativity is often inspired by specific artefacts in the HBB space, which encourages connectivity; #14K describes his meeting table as a place of communication, connection and creativity whilst #24K’s clear desk enables creation of ‘order so your thoughts can flow. . .to start from scratch again for a new project’. #30O’s workspace walls have inspirational quotes, patents, prints, and sketches for his daily work; the cupboard in this space is an example of something he made, which led to new construction projects. Among #33O’s sculptures in his ‘open’ HBB studio is his first piece, which created a pathway out of drug addiction through handcrafting burl-wood. The use of material artefacts makes his ‘open’ HBB space, both for him and those who wish to engage with it, a highly inspirational, creative space.

The HBB is also described by some participants as a lonely space of social isolation despite being punctuated by a degree of connectivity. Those expressing this theme develop tactics to disrupt and resolve feelings of isolation by regular reconnection and social activity. #06K founded a translation forum to relieve feelings of loneliness, where he explained ‘you meet. . .complain and you can also give each other good tips’. We find that these participants accept feelings of loneliness as inherent to their work, often leading paradoxically to self-reflection and calm. #37O’s photograph of camels over her desk leads her to recount how, if working in the evening, she empathises with it – picturing herself like a lone camel when she works late. Participants report experiencing both productive seclusion and isolation. This is illustrated by #17K’s workshop, a space of peaceful seclusion to concentrate on his handicrafts, symbolised by rolled down blinds that signal his wish to work in solitude, which paradoxically, he relinquishes when inviting people into this space or rolling up the blinds for passers-by to look directly into his workshop. Indeed, home is symbolic of a quiet workspace for many of the participants; for #14K, for example: ‘to work on some sunny island, or to sit in a café. . .I can’t do that. Or. . .write texts on the train. . . I just need a quiet place that is *mine* and where I can regather myself. Where can I concentrate’. For #27K, working from home is important and valued as alternative spaces do not provide a similar sense of peace: ‘what I don’t do, because I tried and it just doesn’t work, is go to a cafe or read in a park. . .I need peace so I can concentrate’.

#### Spaces of proximity, safety, security, and support; and of disruption and distraction

Our participants emphasised the spatial proximity of the HBB as convenient and ‘being there on hand’, rendered by spatial immediacy. Participants stress the spatial proximity of the HBB and how much they value its proximal nature; this is contrasted with the ‘terrible experience’ of commuting and long hours away from home (#04K); having to travel in inclement weather (#22K); or having work terms and conditions dictated by others (#30O, #31O, #41O). Spatial proximity for the HBB entrepreneurs is about having easy access to their workspace with all their required equipment, materials and paperwork close at hand (#24K, #37O). For #40O, who previously had business premises 8 KM away, changing this so that this site of production and his home are in spatial proximity ensures he is better able to coordinate home-work activities.

Safety and the linked notion of security were emphasised by #26K and #31O; the former had undergone bankruptcy and numerous health issues, including panic attacks. Her HBB gives her control to manage her conditions on her own terms without any unwanted surveillance or intrusion from others: ‘. . .it’s extremely positive for me to sit here. If I had an order for a website now, it wouldn’t be an issue with the panic attacks either. . . . at home it’s all just OK but not if I’m stuck in an office somewhere’. The latter, #31O, felt her HBB was more secure than an external office; thus, she stored customer data there: ‘My *head-office* was always at home. . .my focus has always been my home. . . having my customer data somewhere else was intolerable for me. I always felt better for having it with me’.

Most participants (19) talk about the support they receive from spouses or partners with non-HBB work, such as childcare and household duties, and HBB-related work, such as business advice, general support, help with initiation of self-employment, IT support, accounts and shared tasks. Traditional gender roles do not appear evident in this respect; these participants did not raise this as an issue; almost an equal number of female spouses (10) and male spouses (8) offered each other support, with examples given of each undertaking various tasks from the list above with the exception of IT support, which was only offered by the men. Support was also linked to notions of social connectivity, shared purpose and community. The latter is illustrated by #06K, a translator, who co-founded a forum for his fellow translators that became a valued space of security and social support to his HBB.

Paradoxically, along with proximity, safety, security, and support, disruption and distraction were also evident. These were experienced in diverse ways with homelife overlapping with work time and the work space; thus, #34O, a naturopathist, after reflecting on both the positives and negatives commented: ‘One of the drawbacks is that sometimes I don’t get any rest even on Saturdays and Sundays, I have people ringing at the door, or patients ringing me up or sending me messages via WhatsApp’. #35O noted: ‘I can be reached 24 hours, 7 days a week, because it is incredibly difficult to remove oneself from it and to say I won’t pick up the phone. . . customers of course already know that’. There is no peace on weekends or holidays as: ‘They call me up on Saturdays, they call me up on Sundays, and there are also some who call me on Christmas Eve. . . I decide whether I will do it or not’. #16K’s strategies involve reclaiming her home in evenings and weekends, restricting client contact, and removing work-related artefacts and items. The participants referred to disruptions to work rhythm, also being affected by neighbours. For #32O ‘. . .one thing I find important, is that the internet connection here is not good. . . when children come home from school. . . when people come home from work, it suddenly becomes very unstable’. Some disruptive forces were not always unwelcome however; this is illustrated by #30O when discussing the simultaneous but contradictory mixed sense of pleasurable annoyance felt when his working day was disrupted by his dog: ‘Sometimes [the dog] comes in somehow and then she barks. She says *Woof*. . .comes closer and ‘Hey’, until I eventually react, and then she jumps at me’.

## Discussion: Theorising entrepreneurial HBB hybrid space – A Triadic Spatial Paradox

**Figure 1.**
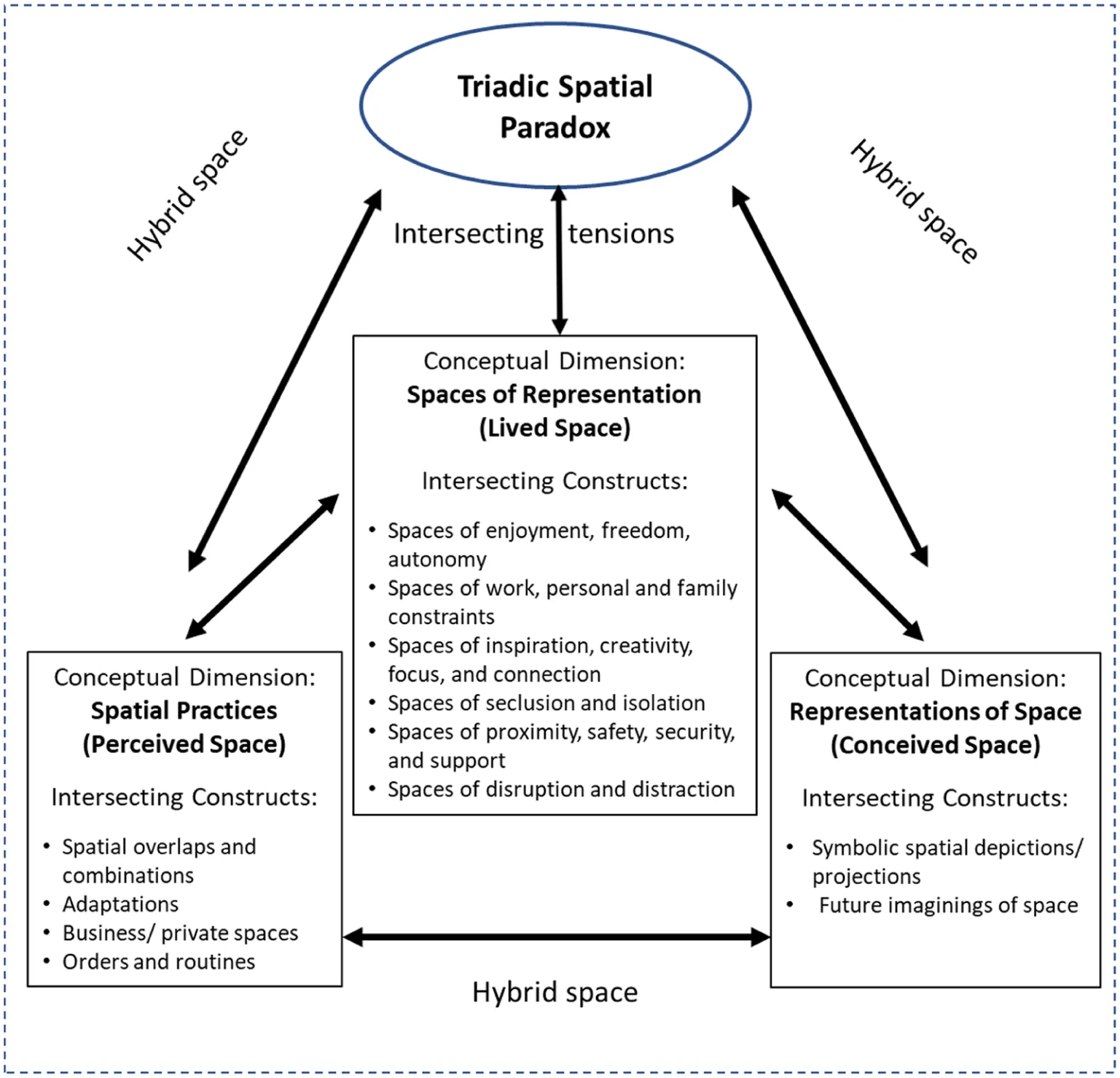
Model of the ‘Triadic Spatial Paradox’ of hybrid-space.

Findings from our analysis enabled us to develop new conceptualisations of the entrepreneur’s relationship with hybrid (home) workspaces, enabling a better understanding of the importance of space in entrepreneurship. In particular, responding to calls for more research in this area, (see Korsgaard et al., 2020), our study enables us to unpack spatiality experiences and how these shape creativity, learning and decision-making in entrepreneurship (Korsgaard et al., 2020; Welter et al., 2017) and particularly, in relation to HBBs. We developed a conceptual model (Figure 1) and the novel notion of the ‘Triadic Spatial Paradox’, revealing an interplay between three Lefebvrian elements which emerged in tandem with paradoxical tensions. Our model enables enhanced theorisation of how the participant entrepreneurs relate to hybrid HBB workspace. It encompasses how this space was viewed and experienced in everyday life (Korsgaard et al., 2020; Trettin and Welter, 2011; Welter and Baker, 2021; Welter et al., 2019) and how multifaceted paradoxical tensions permeated such experiences of the hybrid space as perceived, conceived and lived space. The identified dimensions in these triadic elements illustrate the entrepreneur’s reported perceptions and experiences. These are inductively derived from, and grounded in, their own narratives. We now synthesise our findings to explicate the contribution of our new concept of the ‘Triadic Spatial Paradox’ and our conceptual model (Figure 1).

As reflected in our model (Figure 1) we found spatial hybridity to be an overarching theme; we argue that it permeates all the triadic spatial elements, being particularly notable in the overlapping combinations that these HBB entrepreneurs perceived through mental shifts prompted by their spatial practices that in turn, inspired their creative entrepreneurial imagination (Bjerke and Ramo, 2011; Kier and McMullen, 2018). This adds to the existing literature on hybrid workspaces, given the novel exploration of spatial hybridity for HBB entrepreneurs in terms of their unilocational working practices. This contrasts with the home/external-office multi-locational working, informing previous studies (Halford, 2005; Hislop and Axtell, 2009). To advance the literature, we explore the hybrid socioeconomic mix of values the participants expressed in their ventures, which did not involve social entrepreneurial activities (Shepherd et al., 2019). Our findings show that entrepreneurs use hybrid homespaces for lone work and domestic activities, with these spaces reflecting a hybrid mix evident through overlapping and combined activities. Social values were invested in the home workspaces, with these values reflected back in relations with family, friends and community, as well as business-related entrepreneurial aspirations.

### Spatial practices

The importance of the HBB entrepreneur’s relationships to their home workspace and objects therein is reflected in the spatial practices and processes involved in continually intertwining their social and enterprise practices in this space. Our inductive analysis and findings reveal the dimension of ‘spatial practices’ which constitute various key elements of perceived space as shown in the model (Figure 1), including: spatial overlaps and combinations; adaptations; business workspaces/private homespaces; and orders and routines. Regarding the facet of overlaps and combinations, the paradoxical nature of these elements permeated participant perceptions as the flexibility of a hybrid, primarily unilocational work/homespace is both welcomed and resisted as an invasion of private space. This spatial hybridity denotes the flexibility and deployment of ongoing transformative thinking and mental shifts in spatial perceptions by transformations in spatial practices, but of a different type from that of a multi-locational home/external-office worker (Halford, 2005; Hislop and Axtell, 2009). Ambivalent feelings about how work and home life balance for knowledge workers (Daniel et al,. 2018) and for HBB entrepreneurs (Kapasi and Galloway, 2015; Vorley and Rodgers, 2014) can be seen to extend to HBB entrepreneurial spatial relationships that combine work and homespaces, which are marked by paradoxical tensions that are seen to motivate entrepreneurial creativity.

We ascribe, as part of Lefebvre’s frame of Spatial Practices, ‘adaptations’ to be a key emergent theme advancing research using a Lefebvrian lens (Wapshott and Mallett, 2012). Homeworking can lead to changes in physical space arrangements and home layouts. Yet, spaces are fluid (Nansen et al., 2009, 2010) and can be converted to domestic/work uses. Informed by our interpretive findings and observations, we contend that ‘adaptations’ are affected by HBB spatial practices, which in turn are shaped by the location of the home and its impact on the business, including available amenities, infrastructure, and also the support of family, friends, social networks and the community. This deepens understandings of the complex nature of entrepreneurial homeworking (Daniel et al., 2015; Duberley and Carrigan, 2013; Felstead and Jewson, 2000; Felstead et al., 2001).

As illustrated by our study, business workspaces/private homespaces are integral to an HBB entrepreneur’s experiences of spatial practices. We analysed participant depictions of how and why they used specific physical rooms, zones and boundaries for business/private purposes, despite private spaces often becoming dual-purpose or hybrid ‘re-spatialised’ home workspaces (Halford, 2005). We found that participants emphasised those particular needs that encompass their dual requirements for workspace and homespace, which can be in balance, or in paradoxical opposition (Hargrave and van de Ven, 2017). The ‘home office’, an exclusive space for lone paperwork, computer work and meetings, was the most prominent home workspace. Many, mainly younger city-based entrepreneurs, do lone work in hybrid spaces at home, considering it to be much more social, involving them paradoxically in both isolation and connectivity with clients and household members. It reveals new insights to add to existing research on the importance of social and other networking spaces (Stuart and Sorenson, 2005). Home workspaces for HBB entrepreneurs allow fluidity and malleability in the ‘working home’ (Nansen et al., 2010), which can benefit the business but also create tensions regarding potential invasion of, or perceived encroachment upon, private/family life (Di Domenico, 2008). Part of the endeavour of manoeuvring the hybrid and ambiguous hybrid space is to deploy self-imposed rules that increase the entrepreneur’s feelings of being in control of the space and the other actors therein, such as clients, in terms of permissions and timings of entering and exiting the space. As autonomous entrepreneurs (Vorley and Rodgers, 2014), such individuals can choose to follow these rules if and when desired, or indeed there is personal freedom to alter or break them if they see a need to do so.

HBB entrepreneurs may choose to use external workspaces outside the home; yet, they still depend on the home as the key workspace being linked or ‘tethered’ to the home, just as are the many knowledge-workers whose work is predominantly homebased (Daniel et al., 2018). Even if tethered to the home, this concept means that HBB entrepreneurs are not completely tied physically or mentally to the home; they can still work in external spaces, though they may often choose to order external practices in terms of the strategies they use at home (Hislop and Axtell, 2009). These are then still set by the homespace/workspace rhythm, as controlled by these home-tethered entrepreneurs and can be deployed to help equalise paradoxical tensions arising from work and the home; indeed tethering can be seen as a concept involving both restrictions and also, imaginative ways of working – where home is a central hub but, even if physically tethered to the home, entrepreneurs can experience cognitive freedom to escape the tether by transcending its spatial restrictions and conventional practices. This contributes to the literature regarding how innovative ways of working are conceived, developed and experienced (Cohen, 2010; Daniel et al., 2018; Hill et al., 2003; Kingma, 2016, 2019; Pinelli et al., 2025), and theoretical analysis of how the entrepreneurial imagination can be inspired (Kier and McMullen, 2018).

The entrepreneurs use timed orders and routines to negotiate HBB requirements and privacy. These, though set within the rhythm of the temporalised spaces of everyday life (Lefebvre, 2004; Nansen et al., 2009, 2010), are still imbued with tensions, ambiguity and the interlocking demands and priorities involved in running an HBB. It provides space to connect creative work with the home to relieve work stresses, though there can be a sense of ambivalence by those who struggle to stop working or thinking about it. Although the participants were mostly positive about the spatiotemporal flexibility afforded to them by their HBB (Thompson et al., 2009), they still paradoxically embraced their largely self-imposed business and personal order routines.

### Representations of space

Our findings reveal ‘representations of space’, with constructed elements of conceived space shown in the model as symbolic spatial depictions and future imaginings of space (Figure 1). In terms of symbolic spatiality, entrepreneurs depict the HBB space in their paradoxical mindset to themselves, and to others, representing an entanglement of past/ present/ future, actuality/ potentiality mixes that they positioned in their current creative imagination for a possible future reality. As well as being imbued with dialectical contradictions, as for Lefebvre (2014), this is more transformational; as such, we extend the Lefebvrian frame with a paradoxical lens so as to adopt a perspective and appreciation of representations of space, not just dialectically produced, but more complex, nuanced and irreducible. This illustrates the manner in which Lefebvre’s triad can be regarded less as a form of dialectic, but rather as an arena of nuanced spatial entanglements.

This is how paradoxes develop that we relate to the hybrid home workspace, and what different spatialities reveal to entrepreneurs about future possibilities regarding these spaces. Conceived space for these entrepreneurs emerges in the pictures and layouts of the HBB space that they develop in their imagination, similar to architects planning what they want a space to be like from what it is and has been. Entrepreneurs use symbols to depict the HBB space to both self and others, such as potential and actual clients, where it can be either a self-told story or form part of promoting the HBB’s visibility/attractiveness to prospective customers as a business strategy.

Imaginings of space often involve depictions requiring home workspace adjustments to accommodate plans such as business growth, development, scaling back or retirement. It reflects another level of paradoxical tensions (Lewis, 2000; Smith and Lewis, 2011) that the owners experience for present/future hybrid home workspace use, in attempting to achieve a balance of actualities/potentialities. In effect, they try to manage continual, coexisting, paradoxical tensions (Hargrave and Van de Ven, 2017), and how they relate to them (Daniel et al., 2018). Space-related tensions between lived homespaces and workspaces are potentially resolvable in the future; such tensions and paradoxes coexist in contradiction with synergistic oppositions (Hargrave and Van de Ven, 2017). There is a continuous move to equalise paradoxes (Smith and Lewis, 2011), such as those involving hybrid workspaces with tensions, that can be creatively managed. However, HBB paradoxical tensions in the hybrid home workspace are not easily resolved as they tend to persist in time. Imagined space is imbued with spaciotemporal meanings affecting entrepreneurial social practices (Lefebvre, 2000; Merrifield, 1993). Representation and adornment of such imagined spaces result from HBB entrepreneurial perceived interests, illustrating the Lefebvrian approach to spatiality as multileveled and socially-constructed (Taylor and Spicer, 2007) with spaces infused in social actor self-definitions, values and meanings.

### Spaces of Representation

Within this study, we also found that the dimension of ‘spaces of representation’ constituted elements of lived space. These are shown in the model (Figure 1) as: spaces of enjoyment, freedom, autonomy; spaces of work, personal and family constraints; spaces of inspiration, creativity, focus, and connection; spaces of seclusion and isolation; spaces of proximity, safety, security, and support; and also, spaces of disruption and distraction.

Hybrid spaces deemed by the participants as spatiotemporal spaces of enjoyment and freedom are bound up with flexibility, balancing work with family/homelife. Thus, home workspaces allow flow between work and family (Daniel et al., 2015; Duberley and Carrigan, 2013). Efforts to attain the elusive balance between them are affected by paradoxical constraints from work/ life demands. Entrepreneurs manage tensions while desiring personal fulfilment to enjoy the freedom facilitated by autonomous working, co-situating work and living spaces, and not having to compromise or sacrifice one domain for the other (Vorley and Rodgers, 2014). Enjoyment and freedom are constrained by work, personal and family duties, imbuing home workspaces with inherent ambiguities born of hybridity. The ensuing sense of freedom is enhanced by autonomy and control over business, time and private life, arguably enhanced by imaginatively using hybrid everyday spaces. Yet, these participants continually negotiated/ balanced work, personal, family and other demands. Self-determination and personal validation of their whole self reward their entrepreneurially managing the lived HBB homespace as a dynamic hybrid space in their aim for these ideals.

The HBB as a hybrid space represents an opportunity and vehicle for inspiration and creativity, focus, innovation and imaginative thinking; inspiration and creativity are not necessarily about social interaction, but rather engaging with the HBB space and the presence of material artefacts – and their positions in different spaces in the configuration of the rooms. Paradoxical tensions encourage creative use of home workspace artefacts, bringing to mind de Maistre’s ‘Journey Around My Room’ (1794), and a micro world of inspiration for an individual therein depending also on their own singular subjectivities. As a hybrid space punctuated by connectivities, the HBB is paradoxically still a space of social isolation, along with its benefits and drawbacks (Daniel et al., 2018). Many HBB entrepreneurs accept feelings of loneliness as inherent to their work, leading to self-reflection and, for some, the use of mindfulness to release the deeper creative aspects of entrepreneurial imaginings. Feelings of isolation in the workspace intertwine with co-existing notions of the space as one of highly valued, productive seclusion, autonomy and reflection. As a catalyst of new ideas, it reflects the prescient nature of the hybrid HBB as a space of peace, solitude, privacy, and focus, a quietness conducive to productive work. This experience of productive seclusion and isolation also supports our ideas on the paradox of spatial elements, which are imbued with inherent tensions and co-existing oppositional forces (Hargrave and Van de Ven, 2017). These are at play in the hybrid HBB, which in turn reflects the entrepreneur’s imaginings.

The participants in this study also emphasised that the spatial proximity of both facets of work and home, informing the hybrid HBB, was very convenient due to the inherent intimacy of the space, and its dual function as both work and home. Feeling safe and secure in HBB space links intentionality to feelings of control, authority and agency, and feeling able to order the space, its components and related artefacts. Linked to security, pervading participant representations of HBB space are notions of support, a facet magnified by their significant others and those with whom they feel there is discretion in interacting. This offers a sense of control over spatial interactions in their HBB despite acknowledging that support, or help, with the business can be an inevitable reality for them, given the nature of the HBB workspace. Support links to notions of social connectivity, shared purpose and community. Paradoxically, disruption and distraction co-exist with proximity, safety, security and support, in reflecting the essence of the hybrid HBB space and its competing dimensions of opposing paradoxical forces and tensions (Hargrave and Van de Ven, 2017). When home disrupts worklife, the entrepreneurs tend to engage with, and then employ, their learned flexible abilities in the HBB workspace to imaginatively adjust to their coexisting spaciotemporal pressures. Instead of feeling defeated, they were able to flourish in these paradoxically constructed spaces of different opposing realities, using them to be self-motivated and driven, even acknowledging that disruptions involving resource constraints and technological necessities are beyond their control and unavoidable (Kroeck et al., 2010). Paradoxically, disruptive forces are not always unwelcome as they can provide relief, release, connection or reminder of elements valued as intrinsic to their HBB role. As such, they do not have wholly positive or negative connotations, being more fluid interpretive experiences of space.

## Conclusions and future research directions

This article focuses upon how entrepreneurs personally relate to, and make sense of, the dynamic hybrid home workspace and how they articulate the space-related paradoxes within their HBBs. We drew upon Lefebvre’s triadic framework and, as a novel turn, an intertwined paradox theory lens. Using the Lefebvrian spatial triadic key elements and their rationale as an analytic framework, and combining this with a multifaceted paradox lens, we demonstrate its usefulness for theoretically exploring the HBB entrepreneur’s spatiotemporal experiences. Extant research does not take into account the aspects that we demonstrate are significant by studying the ambivalent, paradoxical relationships that HBB entrepreneurs experience within their highly complex hybrid spaces. This helps advance understandings of everyday entrepreneurship and also, drawing from this study, the spatial context of the home workspace and the paradoxical diversity of HBB entrepreneurial experiences therein. We argue that a Lefebvrian conceptual framework, along with an engagement with the concept of spatial hybridity and a paradoxical theoretical lens, deepens and enhances theoretical understandings of the multifaceted, deeply complex, spatiotemporal HBB experience and its entrepreneurial relationships. We contribute to entrepreneurship research and theorising in relation to spatial hybridity, extending a Lefebvrian framework of space by also exploring the complex, multifaceted paradoxes of this construct – illustrated by our model. Yet, rejecting any implications of a reductive synopsis, the model captures the nuanced conceptual facets that result from our iterative analysis. We acknowledge that the dimensions and sub-elements captured are complex and cross-cutting. They involve an interplay of emerging meanings from a combination of lived, perceived and conceived space and how space actually is, with how it is imagined – which in turn, fuels the HBB entrepreneur’s creativity and the entrepreneurial imagination.

Future research can apply our concept of the ‘Triadic Spatial Paradox’ as per our model (Figure 1), and analytical lens, to alternative hybrid spaces and contexts, including those where dynamic overlaps and tensions pervade the experiences and spatiotemporal perceptions of actors. Future research can further explore the role of performativity and how it is manifest in the HBB, particularly in relation to paradoxes. Implications for practice are to deepen the HBB entrepreneur’s understanding of the importance of their spatial relationships with their work-home business, and concomitant reflective cognizance of other stakeholders, building up heightened awareness and paradoxical mindful negotiation of the hybrid workspace. Further research could also explore in greater depth the concept of ‘tethering’, which we discuss. This could include further research into the evolving nature of self-employment and factors such as diverse generational, gender-related, culturally rooted and other socially or economically based differences in attitudes; for policy decisions to be research-informed and so factor in the increasing prevalence and importance of entrepreneurial HBB owners.
